# Chronotherapy: Circadian Rhythms and Their Influence in Cancer Therapy

**DOI:** 10.3390/cancers14205071

**Published:** 2022-10-17

**Authors:** Ana Amiama-Roig, Eva M. Verdugo-Sivianes, Amancio Carnero, José-Ramón Blanco

**Affiliations:** 1 Hospital Universitario San Pedro, 26006 Logroño, Spain; 2 Centro de Investigación Biomédica de La Rioja (CIBIR), 26006 Logroño, Spain; 3 Instituto de Biomedicina de Sevilla, IBIS, Hospital Universitario Virgen del Rocío, Universidad de Sevilla, Consejo Superior de Investigaciones Científicas, 41013 Seville, Spain; 4 CIBERONC, Instituto de Salud Carlos III, 28029 Madrid, Spain

**Keywords:** chronotherapy, circadian rhythms, cancer, cancer therapy

## Abstract

**Simple Summary:**

Living organisms present rhythmic fluctuations every 24 h in their behavior and metabolism to anticipate changes in the environment. These fluctuations are controlled by a very complex molecular mechanism, the circadian clock, that regulates the expression of multiple genes to ensure the right functioning of the body. An individual’s circadian system is altered during aging, and this is related to numerous age-associated pathologies and other alterations that could contribute to the development of cancer. Nowadays, there is an increasing interest in understanding how circadian rhythms could be used in the treatment of cancer. Chronotherapy aims to understand the impact that biological rhythms have on the response to a therapy to optimize its action, maximize health benefits, and minimize possible adverse effects. In this review, we explore the role of chronotherapy in cancer therapy improvement using different approaches and analyze the effect of administering a therapeutic treatment at a specific and optimal time based on the person’s circadian rhythm.

**Abstract:**

Living organisms present rhythmic fluctuations every 24 h in their behavior and metabolism to anticipate changes in the environment. These fluctuations are controlled by a very complex molecular mechanism, the circadian clock, that regulates the expression of multiple genes to ensure the right functioning of the body. An individual’s circadian system is altered during aging, and this is related to numerous age-associated pathologies and other alterations that could contribute to the development of cancer. Nowadays, there is an increasing interest in understanding how circadian rhythms could be used in the treatment of cancer. Chronotherapy aims to understand the impact that biological rhythms have on the response to a therapy to optimize its action, maximize health benefits and minimize possible adverse effects. Clinical trials so far have confirmed that optimal timing of treatment with chemo or immunotherapies could decrease drug toxicity and increase efficacy. Instead, chronoradiotherapy seems to minimize treatment-related symptoms rather than tumor progression or patient survival. In addition, potential therapeutic targets within the molecular clock have also been identified. Therefore, results of the application of chronotherapy in cancer therapy until now are challenging, feasible, and could be applied to clinical practice to improve cancer treatment without additional costs. However, different limitations and variables such as age, sex, or chronotypes, among others, should be overcome before chronotherapy can really be put into clinical practice.

## 1. Introduction: Circadian Rhythms

Many living beings have a biological clock, the circadian clocks or rhythms. This term, coined by Halberg and Stephens in 1959 [1], explains how rhythmic fluctuations every 24 h in behavior and metabolism help the organism to respond to environmental changes and to organize and/or synchronize different biological and physiological processes to optimize the health of the organism [2]. These rhythms have three basic characteristics: (i) they have endogenous 24-h periods that persist in unchangeable conditions, (ii) they remain in synchronization with the environment, and, (iii) they have a periodicity that does not depend on body temperature [3].

**Figure 1:**
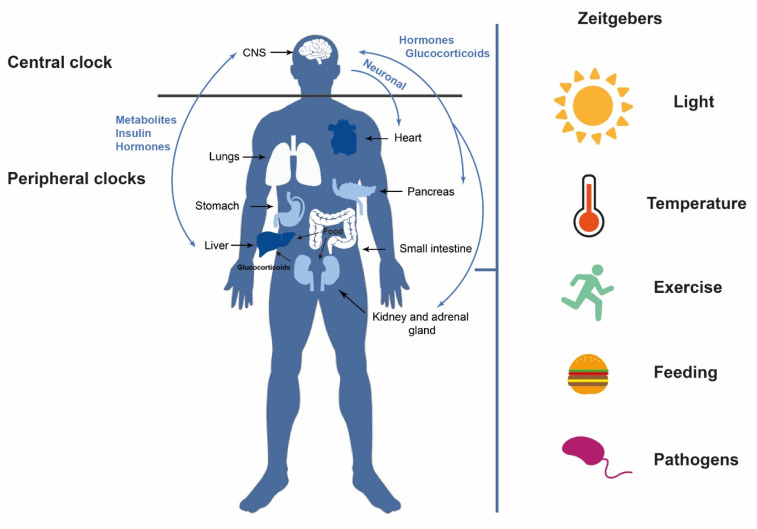
Circadian clocks. The central clock, primarily stimulated by light, communicates with the peripheral clocks and vice versa, through neurotransmitters, neuropeptides, and hormonal secretions. Other external zeitgebers can also influence circadian rhythms such as temperature, feeding, exercise, and pathogens, among others.

Circadian clocks are influenced by the so-called “zeitgebers”, external and internal signals that provide environmental information (time givers) [4]. The most important external zeitgeberg is light, particularly light–dark cycles [5], although there are others such as nutrient availability or temperature [6,7]. All of these signals together modify the expression levels of certain genes and the production of metabolites or hormones that control physiological processes and whose alterations result in damage to health (Figure 1) [8]. An altered clock is a risk factor for multiple chronic pathologies such as neurodegenerative diseases, diabetes, and cancer, among others [9,10]. In addition, this biological clock has important implications for the immune and inflammatory response as well as for tissue regeneration [5]. 

The circadian clock of vertebrates is a hierarchically organized system comprising a network of multiple clocks. The central clock is found in the suprachiasmatic nucleus (SCN) of the hypothalamus, whereas peripheral clocks are located in different tissues and organs (muscle, adipose tissue, liver, pancreas, etc.) [11]. Light is the principal external stimulus and acts by regulating the activity of the so-called circadian pacemaker or central clock [11,12]. The retinal ganglion cells are photosensitive cells that receive visual information from photoreceptors and can transmit the light signal [13]. These cells project to the suprachiasmatic nucleus to maintain circadian rhythms. Therefore, the luminous stimulus promotes the transcription of different clock genes activating the molecular clock [14]. Indeed, each cell is governed by this clock, which comprises approximately 15 genes [15]. The SCN is in turn responsible for keeping peripheral clocks synchronized [13] in other regions inside and outside the brain [3,14] and transmits its rhythmic information through neuronal connections, endocrine signals, and body temperature (Figure 1) [11].

**Figure 2:**
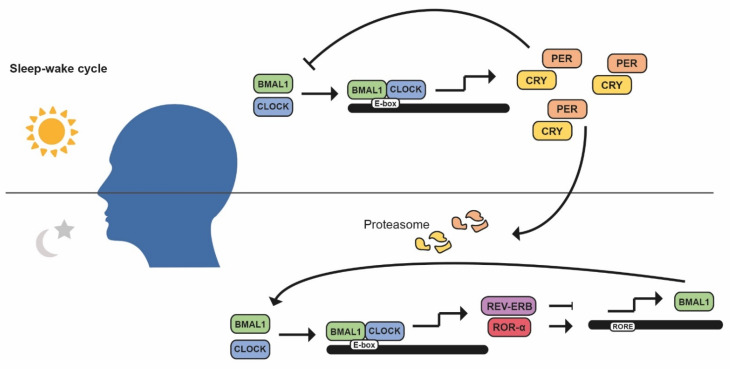
The mammalian molecular circadian clock. Regulation of the main circadian loop (CLOCK/BMAL1) through its activators (RORα) and repressors (PER/CRY, REV-ERB) to control multiple clock-controlled genes.

The molecular clock is orchestrated through by a cell-autonomous system of different autoregulatory feedback loops [3]. The principal loop is composed of the BMAL1 (brain and muscle ARNT-like protein 1)-CLOCK (circadian locomotor output cycles kaput) and PER-CRY (period protein–cryptochrome protein) heterodimers. BMAL1-CLOCK heterodimer is activated during the day and induces PER-CRY transcription through E-boxes, DNA sequences normally located in the promoter region. Throughout the day, PER and CRY accumulate until they reach a limiting concentration, heterodimerize and translocate to the nucleus, binding to BMAL1-CLOCK, repressing their own transcription, and decreasing PER and CRY levels. Throughout the night, PER-CRY is phosphorylated, ubiquitinated, and degraded in the proteasome [2,3]. BMAL1/CLOCK drives the transcription of REV-ERBα, also known as nuclear hormone receptor subfamily group D members 1/2 (NRD1/2), and RORα (retinoic acid response-related orphan receptor α) as their promoters also contain E-boxes. Therefore, a second loop involves both RORα and REV-ERBα, who share DNA binding motifs and compete between them to promote or inhibit, respectively, BMAL1 expression (Figure 2) [2,3,16,17].

Other circadian rhythms independent of gene transcription, as occurs in red blood cells, have no transcription of circadian genes but rather involve rhythmic variations in the oxidation–reduction processes [18]. There is strong evidence that shows the connection between the body clock and the redox status. BMAL1 is implicated in the maintenance of redox homeostasis and cell survival by inducing the antioxidant defenses and protecting cells against oxidative stress. Therefore, the depletion of BMAL1 leads to a premature aging phenotype through an increased oxidative stress [19]. In addition, the expression and activity of some antioxidant enzymes appear to follow circadian patterns [20]. 

An individual’s circadian system is altered during aging and this is related to numerous age-associated pathologies such as metabolic syndrome, cancer, heart disease, neuronal diseases, and an elevated susceptibility to infections, among others [21]. The loss of diurnal rhythms of the immune response occurs during aging and is associated with a disappearance of clock genes transcription in aged macrophages and with smaller chromatin accessibility [22]. The deficiency of BMAL1 during aging is also attributed to a transcription-independent role of BMAL1 in stabilizing heterochromatin [23]. In addition, differences in chronotypes, attending to gender, appear to decrease with age [24].

## 2. Circadian Rhythms and Cancer

At the present time, cancer is the disease that has the greatest impact worldwide in terms of both health and the economy [25]. In 2020, 19.3 million new cases were diagnosed, and more than 10 million deaths were due to cancer [25]. For this reason, it is not surprising that new approaches to cancer treatment should also take into consideration the role that biological rhythms play in the onset, development, and treatment of the disease. In this section, we review different processes related to cancer in which the circadian clock is involved (Table 1).

### 2.1. Cell Cycle Progression

Transitions from one phase of the cell cycle to another are controlled by time windows established by the biological clock, which is known as the circadian gating of the cell cycle. This phenomenon is highly conserved to minimize DNA failures by organizing its replication, for example, during low solar irradiation [26]. Therefore, understanding this circadian gating is of great interest in cancer therapy to optimize the best time of delivery of anti-proliferative drugs [27]. 

**Figure 3:**
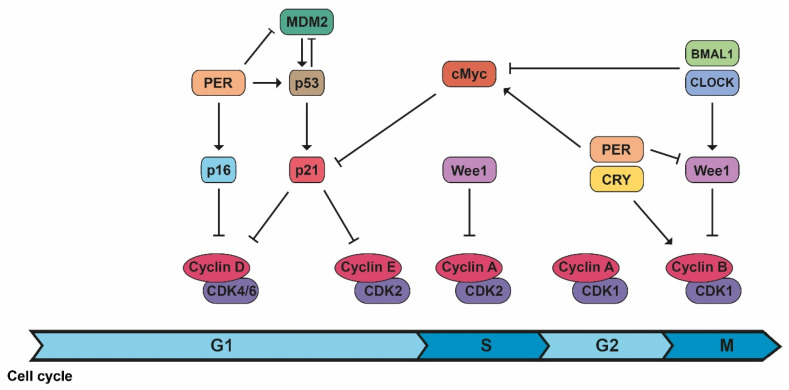
The connection between cell cycle and the molecular circadian clock. The machinery of the cell cycle is controlled by different components of the biological clock in different phases of the cell cycle.

Circadian rhythm control is similar to cell cycle regulation as both are based on periods regulated by transcriptional and translational feedback loops, protein modification, and degradation [2]. The cell cycle is controlled by several highly regulated molecular mechanisms to allow proper cell division: mitosis occurs at certain times of the day in mammals, and some genes controlled by circadian rhythms regulate different phases of the cell cycle [2,3]. CDK1/cyclin B1, the complex responsible for initiating mitosis, is circadian controlled by Wee1, whose expression varies during the day because of CLOCK/BMAL1 activation and PER/CRY inhibition [2,3]. Another protein with circadian activity is PER1, which actually inhibits Wee1 expression. In addition, PER1 interacts with the checkpoint kinase Chk1 and controls the *p16-INK4A* gene, an inhibitor of CDK/cyclin complexes [2]. On the contrary, c-Myc expression is controlled and inhibited by CLOCK/BMAL1 and stabilized by PER1, inhibiting the expression of p21, another inhibitor of CDK/cyclin complexes, and allowing the cell cycle to continue (Figure 3) [3].

In cancer, cell proliferation is increased and circadian clock genes are often severely damped, probably because of mutations in those genes. These altered rhythms also cause a desynchrony between the cell cycle and the body clock [28]. Computational models that take into account this desynchrony are useful to study and understand the effect of circadian patterns of anticancer drug delivery as well as the cytotoxic effect of a drug during the different cell cycle phases [29]. A model for 5-FU and for oxaliplatin found the best time of drug delivery and that the same temporal pattern of administration can have different toxicity towards a cell population, for example, normal versus tumor cells [30]. Computational models allow uncovering new factors that may contribute to improving not only drug tolerance but also efficacy [30]. Therefore, monitoring circadian patterns in cancer patients could offer a new therapeutic tool [31]. However, while the circadian clock might impact the cell cycle, the latter can exist without the former since circadian clock mutant mice exist, do not develop phenotypes associated with cell cycle defects, and are not more cancer-prone [32].

### 2.2. Mechanisms of DNA Repair

Nucleotide excision repair (NER), DNA damage checkpoints, and apoptosis are processes controlled by the circadian clock [33]. Accumulation of DNA damage and failures in the DNA repair mechanisms lead the cell to undergo apoptosis. This programmed cell death is one of our body’s protective systems, as it eliminates cells that accumulate damage. p53, one of the guardian genes of the genome, induces this apoptotic response in response to DNA damage [34]. The clock protein PER2 constitutes a novel downstream effector of the DNA-damage pathway since it binds p53 and MDM2, the p53 inhibitor, to prevent MDM2-induced targeting of p53 to the proteasome. Thus, the downregulation of PER2 affects p53 levels whereas its overexpression influences both p53 protein stability and transcription of p53-targeted genes [35]. In fact, PER2 is regulated by p53, thus all contributing to further control of the cell cycle, the biological rhythm, and the p53-mediated response [3,35]. 

Five main pathways of DNA repair have been described: direct repair, base excision repair, nucleotide excision repair, mismatch repair, and double-strand breaks/recombination repair [33]. Circadian clocks indirectly influence two of them, DNA mismatch repair (MMR) and double-strand breaks (DSBs), as both occur during replication [33,36]. In mice, replication (S phase) of cells mainly occurs in the morning and mitosis (M phase) in the evening [37]. Interestingly, it has been reported that the morning administration of certain genotoxic chemicals (i.e., cyclophosphamide) produces greater toxicity in mice than evening administration [38]. 

**Figure 4:**
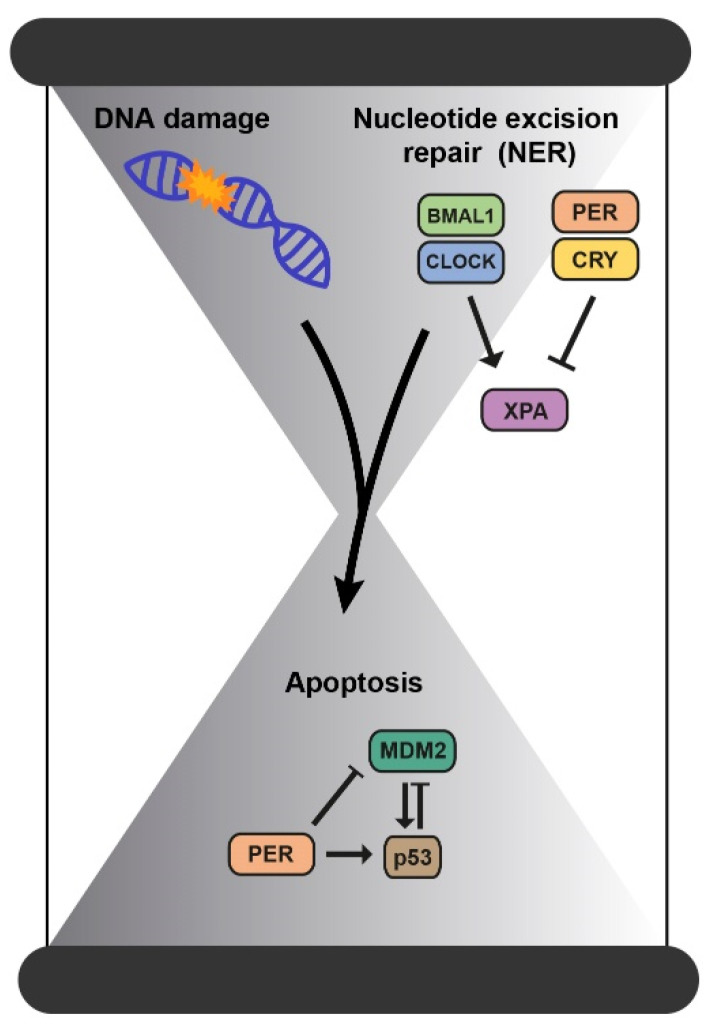
The connection between DNA damage and the molecular circadian clock. Accumulation of DNA damage and failures in the DNA repair mechanisms lead the cell to undergo apoptosis and all these processes are controlled by the circadian clock.

In addition, current evidence shows that only the NER mechanism is directly regulated by the clock through the repair factor XPA [39]. This factor is activated by CLOCK-BMAL1, inhibited by CRY-PER, and implicated in the oscillatory behavior of the repair activity in mice [36,40]. Thus, the maximum peak of NER activity coincides with the maximum peak of XPA, and this determines its repair capacity [36,40]. So, the administration of drugs such as cisplatin in the downstream phase of NER could enhance its therapeutic action at lower doses and therefore be less toxic to the rest of the organism [33] (Figure 4). 

### 2.3. Mitochondrial Dysfunction

One of the milestones that seem to contribute to the appearance and development of cancer is mitochondrial dysfunction. Mitochondria in tumor tissues show higher levels of some reactive oxygen species (ROS), hypoxia, and apoptosis inhibitory signals [41]. Mitochondria present a dynamic process in which they constantly fuse and divide. These changes in mitochondrial morphology appear to be regulated by circadian clocks [42]. In mice knockout of the *BMAL1* gene, low levels of some mitochondrial fusion proteins are observed [43]. Similarly, in those mice lacking PER1/2, mitochondrial respiration is altered [44]. This would imply that disruption of mitochondrial dynamics compromises homeostasis and the health of the organism. Thus, defective mitochondrial function promotes the development of diseases such as type 2 diabetes, obesity, dyslipidemia, and cardiovascular disease [45]. Therefore, the circadian dependence of mitochondrial morphology and its relationship with metabolic and energetic rhythms should be studied and potentially used as a therapeutic target [42]. 

### 2.4. Reprogramming of Metabolism 

Another characteristic of cancer is the reprogramming of metabolism by tumor cells to perform glycolysis and lactic fermentation even under favorable oxygen conditions [46]. This increased glycolysis leads to an accumulation of ROS, DNA damage, and acidification of the microenvironment, which alters the circadian rhythm of the niche in which the tumor cells are located [46,47]. The pancreas is an organ highly influenced by the rhythm of the body due to its role in the metabolism of the organism [3]. During embryonic development, pancreatic differentiation is regulated by the biological clock through Wnt and Notch pathways and the cell cycle [48]. In addition, during the dark period, the exocrine secretion of the pancreas increases [3]. Studies in mice showed that misaligned meals uncouple insulin and corticosterone rhythms in the exocrine part of the pancreas [49]. Thus, unpropitious dietary habits, such as those frequently associated with shift work, may affect the rhythm in the pancreas and contribute to pancreas-associated conditions [49]. Moreover, alterations in sleep habits are associated with high levels of Hemoglobin A1c (HbA1c) in young people with type 1 diabetes and with increased insulin requirements [50]. Therefore, components of the molecular circadian clock are connected to metabolism and could be used as new prognostic factors and for the development of new therapeutic treatments for pancreatic cancer and other types of pathologies [3]. 

### 2.5. The Immune System

Circadian clocks have also been found to be related to the immune system, which shows circadian rhythmicity [51]. For example, in human blood, the highest quantity of undifferentiated T lymphocytes and NK cells is found during the sleep period whereas the lowest is during the activity phase [52]. The production of certain hormones is also controlled by circadian clocks. Regarding cytokine levels, proinflammatory cytokines (such as IL-1β and TNF-α) present their acrophase in the resting period, while anti-inflammatory cytokines (such as IL-4 and IL-10) do so in the active period [52,53]. Another good example is the release of glucocorticoids, potent immunosuppressants, whose peak secretion occurs at the beginning of the day [51]. For example, cortisol levels are higher in the morning and reach their lowest levels during the second half of the night [52,54]. For this reason, hormones could alter the efficacy of immunotherapy treatments in cancer management [55]. The circadian clock works as a gate that controls many processes related to the immune system, including lymphocyte trafficking or antigen presentation, but their impact needs to be further explored. Indeed, some of these biological rhythms are altered in cancer patients [53]. Improving knowledge about the rhythmicity of the immune system could help with developing more effective immunotherapies for cancer treatment as well as with understanding the role that immune cells play in the development, promotion, or death of tumor cells [51].

**Table 1:** Summary of the connections between circadian rhythms and cancer.

**Cell Cycle Progression**		
	Transitions from cell cycle phases are controlled by time windows established by the biological clock.	[26,27]
**CDK/cyclin B1**	Circadian controlled by **Wee1**, whose expression varies during the day because of CLOCK/BMAL1 activation and PER/CRY inhibition.	[2,3]
**CDK/cyclin complexes**	PER1 interacts with the checkpoint kinase **Chk1** and controls the ***p16-INK4A*** gene, an inhibitor of CDK/cyclin complexes. **c-Myc** expression (controlled and inhibited by CLOCK/BMAL1 and stabilized by PER1), inhibits the expression of **p21**, another inhibitor of CDK/cyclin complexes.	[2,3]
**Mechanisms of DNA repair**		
**Mismatch repair (MMR)** **Double-strand breaks (DSBs)**	Indirectly influenced by the clock as both occur during replication.	[33,36]
**Nucleotide excision repair (NER)**	Directly regulated by the clock through the repair factor XPA.	[36,39,40]
**Mitochondrial dysfunction**		
***BMAL1* knockout mice**	Low levels of some mitochondrial fusion proteins.	[43]
***PER1/2* knockout mice**	Altered mitochondrial respiration.	[44]
**Reprogramming of metabolism**		
**Pancreas**	Pancreatic differentiation is regulated by the biological clock through Wnt and Notch pathways and the cell cycle.	[48]
	Misaligned meals uncouple insulin and corticosterone rhythms contributing to pancreas-associated conditions.	[49]
	Alterations in sleep habits are associated with high levels of Haemoglobin A1c (HbA1c) in young people with type 1 diabetes and with increased insulin requirements.	[49]
**The immune system**		
**Sleep period**	Highest quantity of undifferentiated T lymphocytes and NK cells.	[52]
	Highest levels of proinflammatory cytokines (such as IL-1β and TNF-α).	[52,53]
**Active period**	Highest levels of anti-inflammatory cytokines (such as IL-4 and IL-10).	[52,53]
	Glucocorticoids, potent immunosuppressants, peak secretion. For example, cortisol levels are higher in the morning.	[51]

## 3. Chronotherapy: A Promising Therapeutic Option

**Figure 5:**
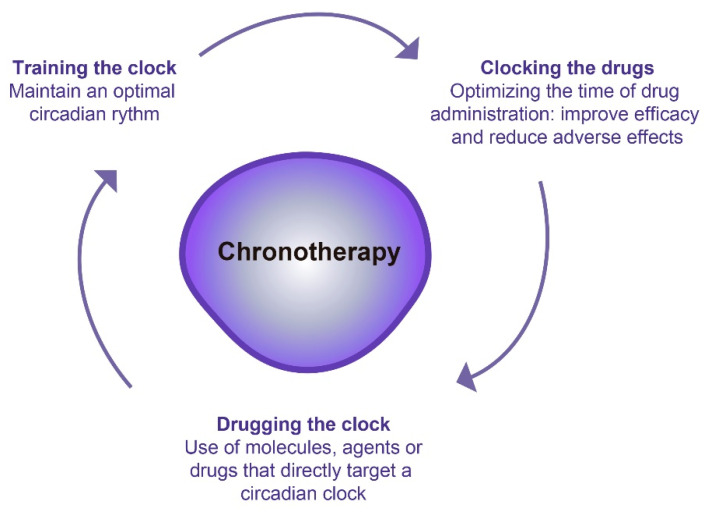
Different chronotherapeutic approaches.

Just as most biological functions are subject to circadian changes [56], pharmacodynamics and pharmacokinetics, are affected by these circadian rhythms [57]. Pharmacokinetics determines the optimal drug concentration needed to produce a balance between efficacy and toxicity. Thus, pharmacokinetic processes are constituted by four different phases: drug absorption, distribution, metabolism, and excretion, known as ADME processes. They show circadian rhythmicity and express different drug-metabolizing enzymes and transporters [58,59], therefore, they can be used in chronotoxicity and chronoefficacy [59]. Nowadays, there is an increasing interest in understanding how circadian rhythms could be used to improve the treatment of different diseases. For this purpose, three different chronotherapeutic approaches have been described, which can be applied alone or in combination. The first encompasses any action that promotes or maintain an optimal circadian rhythm (“training the clock”). The second constitutes the use of molecules or drugs that affect a circadian clock gene (“drugging the clock”). Finally, the third is focused on optimizing the timing and efficacy of drug administration to minimize undesirable side effects (“clocking the drugs”) [60]. Therefore, chronotherapy, or clinical chronopharmacology, study the impact that circadian rhythms have on the response to a drug to optimize its action, maximize health benefits and minimize possible adverse effects on the patient. Thus, chronotherapy consists of administering a drug or applying a therapeutic intervention at a specific and optimal time based on the person’s circadian rhythm (Figure 5 and Table 2) [3].

Phase III randomized clinical trials and meta-analyses [61] that have evaluated the importance of chronotherapy have observed up to 5 times greater tolerability to the drug under study and twice efficacy compared with conventional treatment schedules [15,62]. In this way, we could establish the administration of an anticancer drug at the safest and most effective time [63]. For this reason, circadian clocks could constitute a new therapeutic option. In contrast, other studies comparing the morning and evening administration of a drug have observed no differences in either efficacy or toxicity [64,65]. This variability in results could be attributed not only to the type of cancer and the type of drug but also to the type of comorbidity, genetic (chronotypes) or epigenetic variations, age, sex, etc. [66,67]. Likewise, considering a single time slot would not be a way to address this issue.

Anticancer therapy could take advantage of the mitotic rhythmicity of cells and apply the treatment depending on the time-of-day efficacy [60]. Different factors alter the ADME processes and could modify the action and efficacy of a drug [68]. Therefore, the expression pattern of drug-metabolizing enzymes during the day may be a determining factor in circadian metabolism [69]. Circadian clock proteins control the transcription of these enzymes [70]. For example, BMAL1 regulates the expression of Cyp3a11 during the day, which is involved in xenobiotic/drug metabolism. If Cyp3a11 expression ceases, the toxicity of some drugs is aggravated [71,72].

In view of these results and with the aim of making further progress in personalized medicine, new approaches in the field of chronotherapy are committed to the identification of the circadian rhythms of each individual. In this regard, Martinellli et al. [73] presented the eHEALTH model learning platform. This technology makes it possible to monitor each individual’s circadian markers. Its ultimate goal is to design a method that, based on these circadian biomarkers, allows the prediction of the optimal dose and time of drug administration for each patient. To date, variations in the effect dependent on the time of administration of almost 300 drugs have been documented [74,75], such as amlodipine and many anti-hypertension drugs [76], elobixibat [77], and dexamethasone [78]. 

Another approach to chronotherapy is the development of vehicles that allow synchronization of drug arrival and the circadian rhythm of the therapeutic target. More than half of the drugs act on a circadian gene product [79]. Some pulsatile, osmotic, enteric release or multiparticulate systems can be used for this purpose [8]. An example of this technology, already commercialized, is CODAS (Chronotherapeutic Oral Drug Absorption System), a system that allows drug release with a delay of 5 or 6 h and is used for drugs administered at night [80].

### 3.1. Effect of Chronotherapy in Chemotherapy

Some drugs such as antimitotic agents, antimetabolites, alkylators, or intercalants, usually achieve the best antitumor efficacy when they are administered at the time of the day when they are best tolerated, but this property is not always used for our own benefit [81].

One of the most illustrated examples of how ignoring this property could result in the discard of a useful drug is oxaliplatin. Initially, the development of oxaliplatin was interrupted for undesirable toxicity in a phase I clinical trial [82]. Later, another company studied its safety and efficacy, taking into account chronopharmacology, and they determined that the best way to administrate oxaliplatin is using a chronomodulated delivery that peaks at 16:00 h. The clinical efficacy of oxaliplatin was validated in a large phase II clinical trial in colorectal cancer using this type of delivery and confirmed later in randomized phase III trials [83,84,85,86,87,88,89].

Cisplatin, another platinum analog, has also been studied in chronotherapy. In a study of non-small cell lung cancer by Li et al. [90], no differences were observed in the response to treatment when cisplatin was administered at different times. However, the occurrence of hematological adverse effects such as leukopenia or neutropenia (grade 3 or 4) and gastrointestinal adverse effects (grade 2) after chemotherapy was significantly lower in the group following chronotherapy. Preclinical studies in ovarian cancer patients exhibited that administration of doxorubicin in the morning (06:00) and cisplatin in the evening (16:00–20:00), when both drugs cause less toxicity and tumor response is higher, caused fewer complications and side effects. Indeed, patients treated with this schedule increased their probability of survival at 5 years to 44% [60,81,91]. Similar positive results were obtained when ovarian cancer patients were treated with pirarubicin at 06:00 and cisplatin in the evening [91]. Additionally, this schedule of doxorubicin plus cisplatin was also well tolerated and with a 60% response in patients with advanced/recurrent endometrial carcinoma. In metastatic bladder cancer, this circadian-timed combination chemotherapy also induced a clinical complete response in the majority of patients studied, with an outstanding quality of life and only modest toxicity. Indeed, this regimen also showed a good response as an adjuvant treatment for locally advanced bladder cancer [91].

Fluorodeoxyuridine (FUDR), a chemotherapeutic agent shown to have activity against a variety of malignant neoplasms, can be administered at either a variable or a constant rate. In renal cell carcinoma, continuous and circadian-modulated (68% of the daily dose administered in the evening) administration of FUDR is an effective treatment that induces a durable tumor response with little toxicity [91].

Computational and experimental analysis revealed that the schedule of administration of a given drug could cause different cytotoxicity in the different cell populations. For example, the 5-FU response depends on the oscillation in its target, the thymidylate synthase, and in the enzyme dihydropyrimidine dehydrogenase (DPD), responsible for its degradation. The peak activity of DPD is at 16:00 h, whereas its lowest activity occurs at 4:00 h, which modulates the efficacy of 5FU. On the other hand, glutathione (GSH) is an antioxidant molecule involved in drug withdrawal, and its levels peaked at 16:00 h. It has been reported that some drug toxicities were decreased when those drugs were administered during GSH time of action [60]. In clinical trials of 25 to 35 patients in phase I/II, those patients with digestive cancers who received chronomodulated treatment of 5-FU (alone or with leucovorin), oxaliplatin, or irinotecan, presented fewer adverse side effects [81].

Other clinical trials demonstrated the positive effect of oxaliplatin-5FU-leucovorin treatment through chronomodulated administration in colorectal cancer metastases. Then, to enhance efficacy, two different schedules of administration were designed: chronoFLO4, in which the three drugs were chronoadministered for 4 days with 10 days off, and FOLFOX2, a constant infusion of the drugs for 2 days. In both cases, patients were treated biweekly [88] and they achieved similar survival probabilities with reasonable toxicity. However, the chronoFLO4 scheme produced a survival advantage in males [88]. A meta-analysis corroborated that males lived longer on chronomodulated chemotherapy. Conversely, women had better survival on conventional therapy for localized colorectal cancer than men [92]. Another study showed that irinotecan tolerability was better after morning delivery in men and in the afternoon in women with metastatic colorectal cancer [93]. Therefore, sex is a determinant of better survival and response depending on the drug delivery schedule in patients with metastatic colorectal cancer [92]. This difference is probably because of unidentified differential expression of clock-related genes that control essential cellular processes such as cell cycle progression, apoptosis, mechanisms of DNA repair, and drug pharmacology, which probably makes women respond worse [88]. These findings highlight the necessity to analyze the effect of treatment separately in men and women, as different genotypic and phenotypic profiles have been reported in colorectal cancer. Indeed, women also suffer higher toxicities on 5-FU-based treatment than men, probably because of variations in drug metabolism and detoxification [88,92].

In addition, another study developed a mathematical model that allows personalization of the treatment schedule with irinotecan in colon cancer based on its pharmacokinetics and pharmacodynamics. This model also makes it possible to study the toxicity of the drug according to the levels of expression of genes related to circadian rhythms. Therefore, this model could program the patient’s treatment based on their expression profile and the optimal time to administrate that drug [63]. 

Conversely, it appears that chemotherapy or the administration of some drugs, such as paclitaxel, also disrupts circadian rhythms, the expression of certain related genes, and suprachiasmatic nucleus behavior. All of this supports the idea that therapies based on resynchronizing biological rhythms could improve the living conditions of patients after chemotherapy [11]. In addition, many clock genes could modulate the efficacy of antitumor therapies depending on the time of the day. For example, the DNA alkylator temozolomide and the topoisomerase I inhibitor irinotecan have the maximum toxicity in glioblastoma and colorectal cancer during peak BMAL1 expression [94,95]. Indeed, high expression of *BMAL1* increased the sensitivity to oxaliplatin and paclitaxel in colorectal cancer [96,97].

Clinical trials so far have confirmed that optimal timing of treatment could decrease drug toxicity and increase efficacy, allowing a more dose-intense but successful therapy [91]. Therefore, the combination of chemotherapy with chronotherapy appears to be a promising therapeutic tool.

### 3.2. Effect of Chronotherapy in Radiotherapy

In view of the success of chronotherapy in chemotherapeutic treatments, research has started to look at radiotherapy [98]. However, the application of radiotherapy treatment at different intervals of the day has not been studied in depth. Some circadian genes are involved in the establishment of rhythmicity in the mechanisms induced by ionizing radiation, such as DNA repair or apoptosis, making cells more sensitive to radiotherapeutic treatments at certain times of the day [99]. The radiosensitivity of cells also varies in the cell cycle, being resistant in the S phase and sensitive in late G2/M [100]. Additionally, when cells are replicating they become more radiosensitive, as occurs with tumor cells [99].

A study of brain metastasis in patients with non-small cell lung cancer found a considerable increase in median survival in patients who received radiotherapy before 12:30 h (morning treatment) in comparison with those patients who were treated in the evening [101]. However, another retrospective study of high-grade gliomas showed no differences in the progression-free survival of patients who received morning radiotherapy versus patients treated in the afternoon [102].

Radiation is well known to induce many short and long-term adverse side effects, and its chronomodulated administration attempts to minimize these treatment-related symptoms. Therefore, the aim of chronoradiotherapy focus on symptoms rather than on tumor progression or overall survival [99,103].

A study by Fuzisakki et al. [104] showed that breast cancer patients who received radiotherapy in the afternoon had less skin toxicity than those who received radiotherapy in the morning. Indeed, this effect was stronger in patients homozygous for Per3 and/or for RNA deadenylase Nocturnin (NOC), another clock gene [98]. The individual genotypic profiles appear to be important for the response, as another study in rectal cancer showed increased levels of the circadian genes *CLOCK, CRY2,* and *PER2* in patients on treatment response after radiotherapy [98]. Therefore, it is important to highlight that the individual biological clock has a relevant role in the treatment outcome [98].

As in chronomodulated chemotherapy, there is also a sex dependency in radiotherapy. In a study of bone metastases, only females treated with radiotherapy between 11:00 and 14:00 exhibited a higher complete or partial response [105]. In another study, patients with rectal cancer had a better tumor response when they received radiotherapy treatment after midday, and they suggested a worse response in women [100]. The variability in circadian rhythmicity between women and men could explain this difference in response, but further research is needed in this field [105]. Another variable to take into consideration is age, as was suggested in a study performed on brain metastatic patients. The results of this study demonstrated that overall survival was considerably longer only in elderly women treated with radiotherapy in the morning [106]. However, another study in prostate cancer found that evening radiotherapy leads to worse toxicity and side effects in older patients [107].

Discordances exist in studies that evaluate the chronotherapeutic effect of radiotherapy. Therefore, the possible benefit of this approach should be confirmed in different types of tumors, and in well-designed prospective and randomized trials with proper sample selection [99].

### 3.3. Effect of Chronotherapy on the Blood–Brain Barrier

The most frequent primary brain tumor in adults is glioblastoma (GBM), which presents a very poor prognosis [108]. The standard treatment of this type of tumor consists of surgical resection followed by radiotherapy and administration of the DNA alkylator temozolomide (TMZ) [109,110]. However, the probability of patient survival at 5 years remains very low [110]. Different agonist molecules of REV-ERB (SR9009 and SR9011) and CRY (KL001) have been found to be successful therapies in mouse models of GBM [111,112]. TMZ is capable of crossing the blood barrier readily and presents a short half-life (1.5 h), two characteristics that make it an ideal chronotherapeutic drug. Because TMZ is rapidly absorbed and reaches its highest levels in plasma within 1 h after oral administration, precise dose timing is possible [113]. In a retrospective study, TMZ was shown to increase overall survival when administered in the morning in patients with methylated MGMT (O-6-methylguanine-DNA methyltransferase), the protein that repairs DNA damage induced by TMZ. Indeed, MGMT expression oscillates with the time of the day. Therefore, both MGMT methylation and silencing confer a better response to TMZ treatment [114]. In vitro studies using murine glioblastoma cells showed that administration of TMZ during peak BMAL1 expression in tumor cells can enhance its efficacy [94]. Indeed, preclinical analyzes have observed maximal TMZ efficacy when the application of the treatment coincided with the peak of BMAL1 expression. Therefore, morning TMZ administration appears to be the most effective thanks to its daily oscillations in the absorption/excretion and the sensitivity of tumor cells to DNA damage [114]. Thus, BMAL1 has an important function in the regulation of the DNA damage response, as observed in other studies on colon cancer sensitivity to irinotecan and oxaliplatin [94].

Bortezomib, an inhibitor of the proteasome, is commonly applied in clinical trials in advanced phases of GMB. In vivo studies showed that the use of bortezomib at a low dose did not induce major side effects. Besides, its administration at night was significantly more effective, inducing tumor growth inhibition near 70% in comparison with 18% inhibition of day administration. Therefore, night administration of bortezomib offers a time frame of high efficacy that coincides with when mice are metabolically active [115].

### 3.4. Effect of Chronotherapy on the Immune System

Clock components are also a potential target for immunotherapy and two strategies could be followed: drug development for circadian clock targets and chrono-immunotherapy [53]. 

For the first strategy, different components have been developed. Two RORγ synthetic agonists, LYC-53772 and LYC-54143, which can activate BMAL1 transcription, induce T cells differentiation, block regulatory T cell-induced immunosuppression and elevate the secretion of cytokines [116]. In addition, the treatment with RORγ agonists induced resistance to PD-L1 inhibition in T cells, which eliminate anti-tumor immunity [117]. They also increase the cytotoxic activity of T cells, enabling the regression of tumors in mice [117]. LYC-55716 is another RORγ agonist that has shown initial success in a clinical trial in phase I of locally advanced/metastatic solid tumors of different origins [118]. Besides, a similar clinical trial using this agonist in combination with the monoclonal antibody pembrolizumab in patients with non-small cell lung cancer is in progress [51]. Moreover, the RORα synthetic agonist SR1078 increased CD8+ T cell response to anticancer immunity role [119].

In the case of chrono-immunotherapy, the efficacy of the drug seems to be more relevant under certain experimental and/or clinical conditions. For example, the antitumor effect of interferon-β in mice was more evident during the day instead of at night [120]. In a clinical trial of renal cell carcinoma in phase I-II, IL-2 chronotherapy showed activity and the intravenous infusion was feasible in a standard care unit [121]. Interestingly, a study in patients with advanced melanoma demonstrated that morning or early afternoon administration of different immune checkpoint inhibitors (such as ipilimumab, nivolumab, or pembrolizumab) extended patient overall survival in comparison with late afternoon or evening treatment. Once again, it seems that a more effective immune antitumor response is induced in the daytime in comparison with the evening [122]. A pilot study supported these data by showing that non-small cell lung cancer patients in stage IV who received nivolumab morning treatment significantly reduced their risk of progression and increased their survival [103].

On the other hand, disrupted cortisol expression is linked to tumor suppression. For example, in ovarian cancer, abnormal cortisol rhythms were associated with decreased survival and increased inflammation [54]. In this regard, studies with animal models have demonstrated that high glucocorticoid levels are associated with a reduction in the efficacy of chemotherapy and anti-PD-L1 [123]. Retrospective clinical data also propose that the use of corticosteroids has detrimental effects on anti-PD-L1 response [124,125]. Nevertheless, further studies that clarify the role of corticosteroids in the response to treatment of patients are needed. 

### 3.5. Other Uses of Chronotherapy in Cancer

Other ways to take advantage of the benefits of chronotherapy and synchronize daily rhythms could be light exposure in the morning and/or taking melatonin before sleep [114]. Indeed, a link between melatonin and cancer has also been observed and Li et al. [126] discussed the possible oncostatic impact of melatonin on various types of tumors, such as breast, prostate, gastric and colorectal. This action could be due to its antioxidant activity, stimulation of apoptosis, or inhibition of angiogenesis and tumor metastasis, among others [127]. In addition, Önder et al. demonstrated that treatment with melatonin increased its repressive effect on the growth of breast cancer cells by inducing cell death in vitro [128]. Therefore, melatonin could be applied as an adjuvant treatment to chemotherapy and radiotherapy, making tumor cells more sensitive to both treatments [126,129]. Moreover, those patients that have been exposed to light during nighttime presented a reduced melatonin secretion and an increased incidence of tumor development [130]. 

Certain nutritional behaviors also appear to affect circadian rhythms. For example, caloric restriction, an anti-aging dietary practice, reversed the circadian and metabolic profiles of aged mice [131]. This caloric restriction is also able to reduce tumor progression and to promote tumor cells death, therefore, making antitumoral therapies more effective [132]. However, as caloric restriction also has detrimental effects, other studies have considered intermittent fasting as an alternative, suggesting that this intervention could not only prevent tumor development but also improve the antitumor response of the immune system and the sensitivity to antitumoral therapies. Therefore, a well-designed chronodietary intervention could be a promising therapeutic option against cancer [133,134].

Additionally, it would be of great interest to analyze whether the timing of exercise has an influence on cancer progression and the therapeutic response of patients [60].

**Table 2:** Summary of some chronotherapeutic approaches applied in cancer therapy.

**Effect of Chronotherapy in Chemotherapy**		
**Oxaliplatin**	Chronomodulated delivery: peak at 16:00 h.	[83,84,85,86,87,88,89]
**Cisplatin**	**Non-small cell lung cancer:**	
	Low hematological and gastrointestinal adverse effects in the group following chronotherapy.	[90]
**Cisplatin + doxorubicin or pirarubicin**	**Ovarian cancer:**	
	Cisplatin in the evening 16:00–20:00) combined with doxorubicin or pirarubicin in the morning (06:00) cause less toxicity/side effects and high tumor response.	[60,81,91]
	Cisplatin + doxorubicin had also tumor response in **endometrial carcinoma** and **bladder cancer**.	[91]
**Fluorodeoxyuridine**	**Renal cell carcinoma:** Circadian-modulated (68% of the daily dose administered in the evening) administration induces a durable tumor response with little toxicity.	[91]
**5-FU**	Fewer adverse side effects in **digestive cancers.** Chronoadministration of oxaliplatin-5FU-leucovorin **(ChronoFLO4)** produced a survival advantage in males with **colorectal cancer**.	[88]
**Irinotecan**	Better tolerability after morning delivery in men and in the afternoon in women with metastatic **colorectal cancer**.	[93]
**Effect of chronotherapy in radiotherapy**		
	**Brain metastasis in patients with non-small cell lung cancer:** Better survival in patients treated in the morning (before 12:30 h).	[101]
	**High-grade glioma:** No differences in survival.	[102]
	**Breast cancer:** Radiotherapy in the afternoon induced less skin toxicity.	[104]
	**Bone metastases:** Females treated with radiotherapy in the morning exhibited a higher complete or partial response.	[105]
**Effect of chronotherapy on the blood–brain barrier**		
**Temozolomide (TMZ)**	Morning administration increases overall survival in patients with methylated MGMT, coinciding with the peak of BMAL1 expression.	[114]
**Bortezomib**	Night administration induces 70% tumor growth inhibition.	[115]
**Effect of chronotherapy on the immune system**		
**LYC-53772 and** **LYC-54143**	**RORγ synthetic agonists:** Activate BMAL1 transcription, induce T cells differentiation, block regulatory T cell-induced immunosuppression, elevate the secretion of cytokines, induced resistance to PD-L1 inhibition in T cells, and increase the cytotoxic activity of T cells.	[116,117]
**SR1078**	**RORα synthetic agonist:** Increases CD8+ T cell response.	[119]
**Interferon-β**	Better antitumor effect during the day in mice.	[120]
**Ipilimumab, Nivolumab, or Pembrolizumab**	**Melanoma:** Morning or early afternoon administration extended overall survival.	[122]

## 4. Challenges and Prospects for the Future of Chronotherapy

Within the efforts of the scientific community to advance in the field of personalized medicine, chronotherapy is here to stay. Different studies focusing on the metabolites of the circadian clock have been able to corroborate the 24-h periodicity in the circadian rhythms of most tissues and metabolic processes [135,136]. Potential therapeutic targets within the molecular clock have also been identified [137].

However, for the time being, improving the efficacy and reducing the toxicity of drugs according to the time of administration poses some difficulties. Kuo and Ladurner [138] summarize the challenges of chronotherapy from three perspectives: (a) to design clinical trials that allow a deeper understanding of the effect of biological clocks on the action of anticancer drugs and vice versa; (b) to study the impact that some variables, such as age, sex, comorbidities, etc., have on the response to chronotherapy; and (c) to encourage pharmaceutical companies to evaluate the efficacy and toxicity of their drugs according to chronotherapy criteria. In addition, to guarantee that drugs are released at the right time frame, more efficient delivery systems (such as nanoparticle systems) should be designed and optimized [139].

There are other limitations to the application of chronotherapy, such as the presence of chronotypes. Thus, each person has individual characteristics in the rhythmicity of the different phases [138]. To objectively determine the circadian phase, DLMO (dim light melatonin onset) is used [140]. This noninvasive test consists of obtaining saliva samples from the patient in which the levels of melatonin are measured [141]. Light impedes melatonin secretion, so measurements are taken every 30 to 60 min for 6 to 8 h under very dim light conditions [142]. However, both the price of the test and its duration make this test very complicated to perform at present. Other approaches also propose to use blood tests to determine rhythms from the sequencing of blood. Moreover, Wittenbrink et al. developed a simple but precise assay (called BodyTime) capable of estimating the internal circadian time in humans by measuring some clock biomarkers such as NR1D1, NR1D2, CRY1, PER1, PER2, PER3, CRISPLD2, KLF9, LGALS3, ELMO2, FKBP4, and HSPH1, from a single blood test [143]. 

Recently, great importance has been attached to mathematical models describing tumor growth that consider the possible influence of some environmental factors on the response to treatment [144,145], which is known as control theory [146]. Specifically, there is a growing interest in the application of control theory in models of pharmacokinetics and pharmacodynamics for antitumor therapies. The increasing availability of data from tumors allows the development of mathematical models that predict optimal therapy delivery parameters to control the response to treatment and the time to progression [146]. However, considerable research in this field is still needed before it can really be applied. 

## 5. Conclusions

Circadian rhythms play a key role in the organism, and their alteration is associated with aging and the appearance of numerous age-associated diseases, such as cancer. Chronotherapy is emerging as a new anti-cancer therapy that can improve the treatment of each patient in a personalized manner. In the case of chemo or immunotherapies, optimal timing of treatment could decrease drug toxicity, and increase drug efficacy and tumor response. Instead, the chronoradiotherapy seems to minimize treatment-related symptoms rather than tumor progression or patient survival. Therefore, results of the application of chronotherapy in cancer therapy until now are challenging, feasible, and could be applied to clinical practice without additional costs. However, different limitations and variables such as age, sex, or chronotypes, among others, should be overcome and further studies are required before chronotherapy can really be put into clinical practice. In this regard, mathematical models of pharmacokinetics and pharmacodynamics for antitumor therapies could help to predict optimal delivery parameters and improve the response to treatment, but considerable research in this field is still needed.
